# Reliability of transcranial magnetic stimulation and H-reflex measurement during balance perturbation tasks

**DOI:** 10.3389/fphys.2022.957650

**Published:** 2022-10-14

**Authors:** Nijia Hu, Janne Avela, Dawson J. Kidgell, Samuli Nevanperä, Simon Walker, Jarmo M. Piirainen

**Affiliations:** ^1^ NeuroMuscular Research Center, Faculty of Sport and Health Sciences, University of Jyväskylä, Jyväskylä, Finland; ^2^ School of Primary and Allied Health Care, Department of Physiotherapy, Monash University, Melbourne, VIC, Australia

**Keywords:** dynamic balance control, voluntary activation, motor evoked potential, intraclass correlation coefficients, corticospinal modulation

## Abstract

Following ankle movement, posterior balance perturbation evokes short- (SLR ∼30–50 ms), medium- (MLR ∼50–60 ms), and long-latency responses (LLR ∼70–90 ms) in soleus muscle before voluntary muscle contraction. Transcranial magnetic stimulation (TMS) and Hoffmann-reflex (H-reflex) measurements can provide insight into the contributions of corticospinal and spinal mechanisms to each response. Motor evoked potential (MEP) and H-reflex responses have shown good reliability in some dynamic muscle contraction tasks. However, it is still unclear how reliable these methods are in dynamic balance perturbation and corticospinal modulation during long amplitude balance perturbation tasks. 14 subjects completed two test sessions in this study to evaluate the reliability of MEPs, H-reflex, and corticospinal modulation during balance perturbation. In each session, the balance perturbation system operated at 0.25 m/s, accelerating at 2.5 m/s^2^ over 0.3 m displacement. MEPs and H-reflexes were elicited in the right leg soleus muscle at four delays after ankle movement (10 ms, 40 ms, 80 ms, and 140 ms), respectively. Test-retest reliability of MEP and H-reflex amplitudes were assessed *via* intraclass correlation coefficients (ICC) both between- and within-session. Between-session test-retest reliability for MEPs was excellent (ICC = 0.928–0.947), while H-reflex demonstrated moderate-to-good reliability (ICC = 0.626–0.887). Within-session reliability for both MEPs and H-reflex was excellent (ICC = 0.927–0.983). TMS and H-reflex measurements were reliable at different delays after perturbation between- and within-sessions, which indicated that these methods can be used to measure corticospinal excitability during balance perturbation.

## Introduction

Human standing balance control is defined as maintaining the stability limits between the center of mass and base of support (Maki and Mcilroy, 1997). In dynamic balance tasks, the human center of mass is led to more challenging conditions, in which the somatosensory system plays a more crucial role in selecting an appropriate muscle response for maintaining balance (Horak et al., 1990). When a sudden and unexpected posterior perturbation occurs, the movement at the ankle joint leads to muscle stretch within the shank, which evokes complex reflexes with short- (SLR ∼30–50 ms after ankle plantarflexion), medium- (MLR ∼50–60 ms), and long-latency responses (LLR ∼70–90 ms) (Taube et al., 2006; Latash and Zatsiorsky, 2015). SLR has been demonstrated to be elicited by a pure monosynaptic response at the spinal level, while LLR is influenced more by the transcortical loop, which has been suggested to include supraspinal level involvement since it has enough time to exert its influence (Taube et al., 2008). As perturbation amplitude increases, there is greater time for body sway that predicts more voluntary activation involved to maintain body balance. Stronger calf muscle voluntary contraction ability is related to better balance control that is observed more in young people who use an ‘ankle strategy’ than in e.g. older adults who more often use a ‘hip strategy’ to maintain balance during perturbation (Horak et al., 1992). It has been suggested that both supraspinal and spinal level mechanisms may be at play during a balance perturbation task. However, corticospinal and spinal excitability modulation of responses and voluntary activation during the balance perturbation task is still not clear with higher amplitude perturbation and such studies have been limited.

Transcranial magnetic stimulation (TMS) and Hoffmann’s reflex (H-reflex) measurements are commonly used to induce involuntary responses and study the role of corticospinal and spinal excitability as well as their modulation during various tasks (Pinniger et al., 2001; Trimble and Koceja, 2001; Knikou, 2008). In TMS measurements, a significant practical challenge faced by researchers is the stabilization of the TMS coil during the experiment, which may be more precise by using a TMS navigation system particularly in static conditions. For now, only a small number of studies have used TMS in anterior and posterior balance perturbation, and the maximum perturbation amplitude is 15 cm (Taube et al., 2007; Wälchli et al., 2017; Fujio et al., 2019). A higher amplitude balance perturbation may lead to larger and faster body swaying, which may result in unexpected movement of the TMS coil. Thus, the stability of the TMS coil is critical during TMS experiments, especially in the absence of a neuronavigation system (Chipchase et al., 2012). Stabilization of the TMS coil should be carefully considered when examining dynamic balance tasks. Further, the motor evoked potential (MEP) elicited by TMS is very sensitive to changes in the environment outside of the body (i.e., environment noise) and inside (i.e., awareness switch) (Chipchase et al., 2012). Therefore, testing reliability and variability of MEPs are also crucial within this setting. Many studies have observed acceptable reliability of using TMS in static and dynamic conditions, such as in relaxed muscle, knee contraction, and squat tasks (Tallent et al., 2012; Proessl et al., 2021). However, better reliability has been observed in static tasks (i.e., isometric knee extensions) compared with dynamic tasks (i.e., squats) (Proessl et al., 2021), which suggests that complex tasks with extra technical and physiological noise are more variable when using TMS.

H-reflex measurement has been used to assess spinal (motoneuron pool) excitability (Táboríková and Sax, 1968). Good reliability has been observed in many studies (Hopkins et al., 2000; Hayes et al., 2009), for example, when measuring H-reflex at rest, excellent test-retest reliability was observed in soleus and tibialis anterior muscles (ICC >0.9) (Palmieri et al., 2002; Hayes et al., 2009). Good reliability was also shown in ankle plantarflexion and dorsiflexion positions during isometric contraction and walking in soleus muscle (Chen et al., 2010; Simonsen and Dyhre-Poulsen, 2011). However, increased variability in reliability values was observed in different sitting postures (e.g., erect sitting, slumped sitting, and slouched sitting), while the overall reliability of H-reflex was still good (ICC >0.8) (Al Amer et al., 2020). In summation, H-reflex has been demonstrated to have good reliability during various tasks, but not yet during dynamic balance perturbation trials.

Currently, the reliability of neither TMS nor H-reflex measurement during high amplitude balance perturbation tasks is known, but such methods are used by researchers to examine differences between groups and/or the effect of interventions (Taube et al., 2007; Fujio et al., 2019). Thus, it is important to determine such reliability to enable full evaluation of the scientific methodology employed within those studies. Also, in a previous study, the TMS coil was held by the halo vest on the subject’s shoulder (Taube et al., 2006), but the vest may affect the natural body movement during balance perturbation. In our system holding the TMS coil, the entire coil is connected with the platform, which helps the TMS coil move with the balance platform during perturbation. Therefore, the aim of the present study was to examine the test-retest reliability of MEPs and H-reflex responses as well as corticospinal modulation during a high amplitude balance perturbation task.

## Methods

### Subjects

Fourteen voluntary subjects participated in the study (8 males, 6 females, age: 35 ± 6 years, height: 173.5 ± 10.6 cm, weight: 71.8 ± 17.0 kg, and BMI: 25.0 ± 4.7). None of the subjects had any history of neuromuscular or orthopedic diseases and all subjects were informed about the procedures and gave written informed consent. Subjects were fully introduced to the protocol and they had the opportunity to withdraw from the study at will in any phase. The study was approved by the ethics board of the University (diari number: 1267/13.00.04.00/2021) and the study was performed in conformity with the declaration of Helsinki (2013).

### Experimental design

**FIGURE 1:**
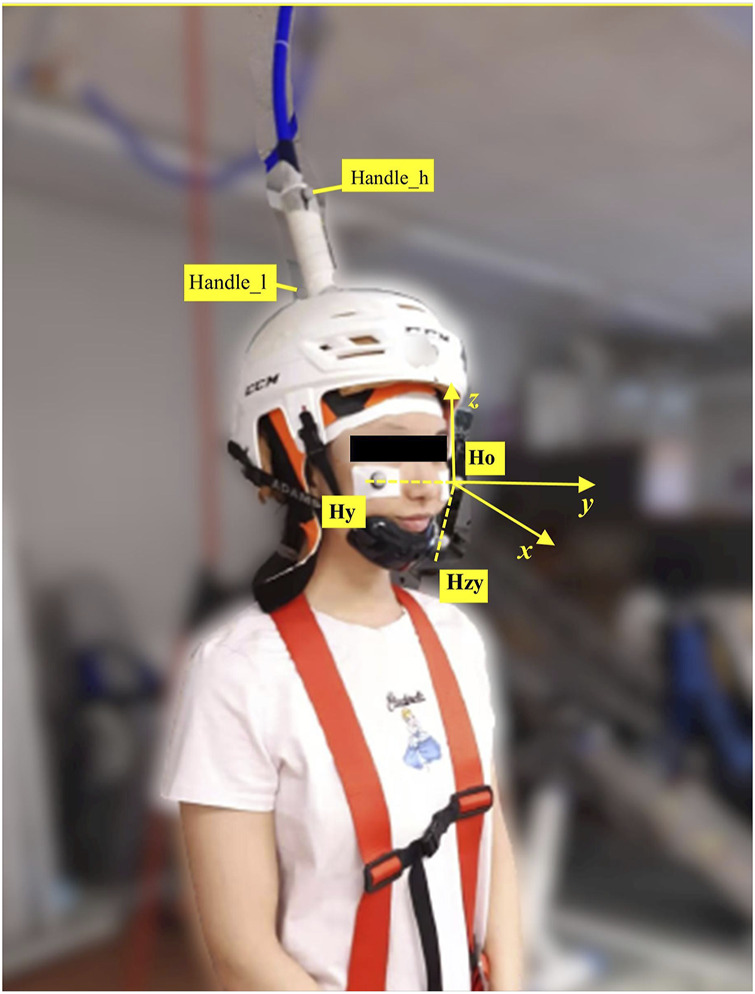
Motion capture markers were placed as shown in the picture. Three markers were placed on the head, i.e., ‘Ho’ was the origin of the head coordinate system; ‘Hy’ was utilized to build y-axis with ‘Ho’; ‘Hzy’ was the point on the zy-plane, which produced *x*-axis by cross product with *y*-axis). *z*-axiz was built by cross product of *x*-axis and *y*-axis. Handle_h was the marker on the higher position of the TMS handle, and Handle_l was the marker placed on the lower position of the TMS handle.

Tests were conducted over two sessions with the same tasks repeated and 46 ± 7 h separated Session 1 (S1) and Session 2 (S2). In each session, after electromyography (EMG) electrode setup and 5 min cycling warm-up (70W) on the fitness cycle (Monark, 282E, Varberg, Sweden), 16 balance perturbations without any stimulation were used to collect center-of-pressure (COP) and EMG activity data. Then, subjects were positioned in a custom-built ankle dynamometer (University of Jyväskylä, Jyväskylä, Finland) to test the isometric maximal voluntary contraction (IMVC) of the right leg. The TMS coil was set up and the active motor threshold (aMT) was tested when subjects sat in the ankle dynamometer. With a TMS coil set on the head and held by the custom-built helmet (Figure 1), subjects carefully stood up and moved to the balance platform. In the balance perturbation task with stimulation, MEPs were evoked at four different delays after the onset of ankle movement during balance perturbation in random order. The H-reflex measurements were always performed after TMS due to practical reasons. H-reflexes were elicited at the same four delays as the MEPs also in random order. The stimulations were delivered during each balance perturbation, regardless of perturbation direction, but only MEPs and H-reflex during backward perturbations were analyzed.

### Pre-study design

A pre-study experiment was performed with two subjects with different height and weight before the main experiment to investigate the stability of the custom-built TMS coil helmet and TMS cable holder system. Kinematic data of the TMS coil and the head of the subject were recorded at 150 Hz by a five-camera motion capture system (Vicon Motion System, Oxford, United Kingdom). Three markers were placed on the subject’s head to build the head coordinate system. Two markers were placed on the coil handle to estimate TMS coil movement since the coil was totally covered by the helmet, which made it impossible to place any markers on the coil itself. Kinematic data were analyzed using MATLAB (2019b). After coordinate transformation from ground coordinate to head coordinate system (see Figure 1), relative offset (maximum displacement) of the coil handle was analyzed to represent coil movement compared with the subject’s head movement. The *x*-axis was the sagittal axis, the *y*-axis was the frontal axis, and the *z*-axis was the vertical axis.

### Electromyography

EMG was measured by bipolar electrodes (Blue Sensor, Ag/AgCl, 28 mm^2^, Ambu A/S, Ballerup, Denmark) placed 2 cm below the gastrocnemius on the line of the Achilles tendon for soleus muscle (SOL) and tibialis anterior (TA) and gastrocnemius (GM) muscles according to SENIAM guidelines (Hermens, 1999). As part of TMS measurement, we used the pseudo-monopolar setup to collect the MEPs considering potential discomfort and intension of subjects caused by high intensity stimulation during balance perturbation, especially during 140 ms (voluntary activation phase). The pseudo-monopolar setup allowed MEPs of higher amplitude to be recorded compared with bipolar connection, which in turn also decreased the intensity of the stimulus needed to evoke a detectable MEP (Kirk et al., 2019). According to our practical experience, the shape of the MEP is more consistent with the pseudo-monopolar setup, which is important for the dynamic task*s*. A disadvantage of this electrode montage is that the signal-to-noise ratio can be compromised. However, this was not a problem in the current setup. One electrode was placed 2 cm below the gastrocnemius on the line of the Achilles tendon and the reference electrode was placed on the tibia at the same level. The skin was shaved, carefully abraded with sandpaper, and cleaned with alcohol. Skin target impedance was less than 5 kΩ and if this was not the case, skin preparation was repeated. All EMG data were collected using the Neurolog EMG system (CED ltd., Cambridge, England), with a gain of 1000. Data were band-passed (15–500 Hz) filtered and further collected using CED 1401 A/D-converter (CED ltd., Cambridge, England) and Spike 2 (8.0) software (CED ltd., Cambridge, England) with a sampling rate of 5 kHz.

### Isometric maximal voluntary contraction

Isometric maximal voluntary contraction (IMVC) was used to investigate possible muscle fatigue between sessions and to measure aMT. After EMG setup and a 5 min warm-up, subjects were positioned in a custom-built ankle dynamometer (University of Jyväskylä, Jyväskylä, Finland) to test the IMVC with the right foot on the plate at 100° hip angle, 180° knee angle (leg fully extended) and 90° ankle angle. After the positioning procedure, the subject contracted 5 - 7 submaximal plantarflexion trials to practice the performance. IMVC was performed at least three times at one-minute intervals and the highest force value was considered as the IMVC. If the last trial was >5% higher than the second-best, single additional trials were performed until no further improvement was observed. The typical number of required maximum trials was 3–5. Reaction forces from the dynamometer pedal were measured and maximum IMVC amplitude was analyzed by a strain gauge transducer sampled at 1 kHz in Spike2 software.

### TMS and H-reflex measurement setup

**FIGURE 2:**
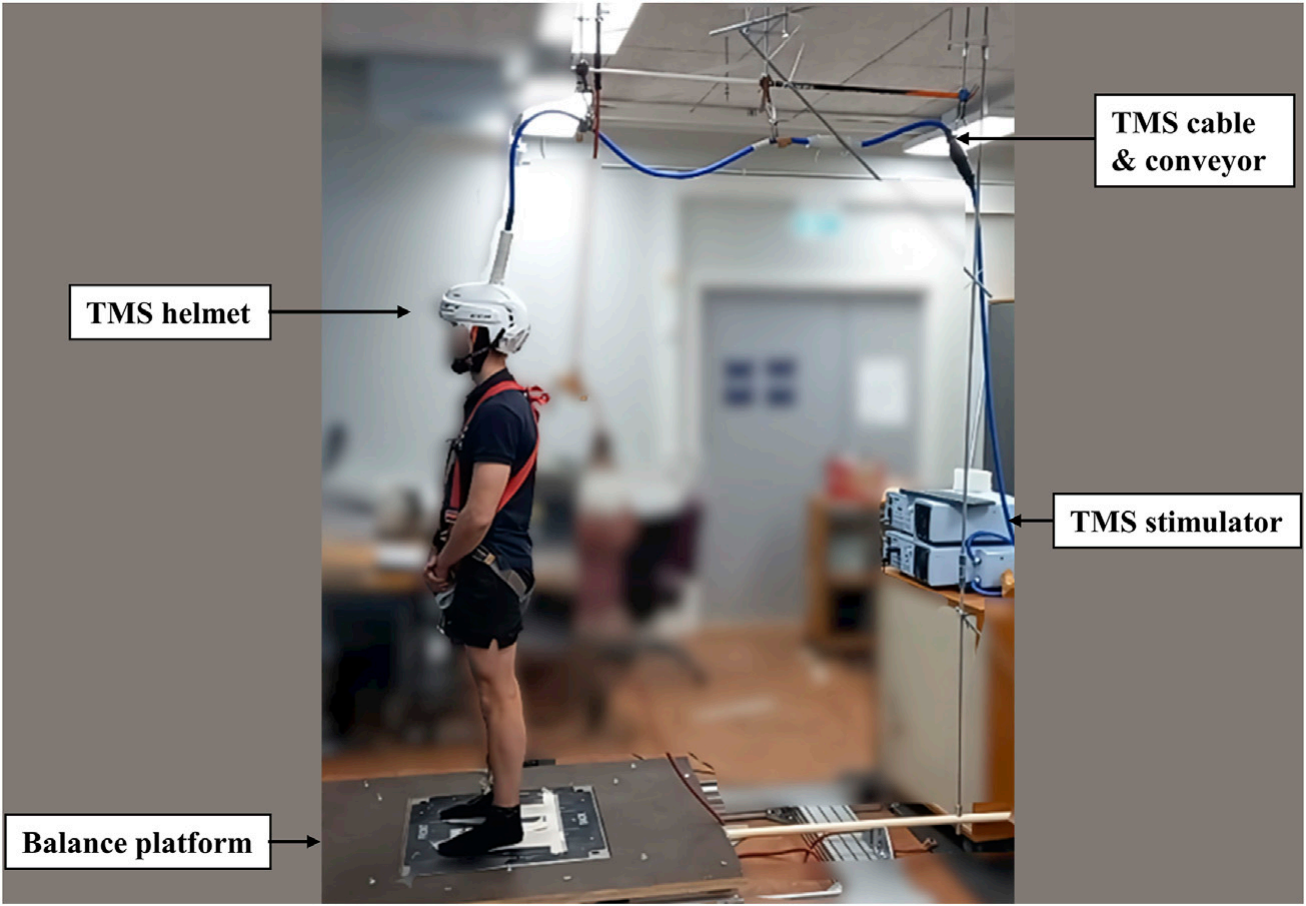
The figure shows the modified helmet to stabilize the TMS coil. The TMS coil’s cable was connected with a conveyor on the roof to relieve the weight and moved along with the balance platform during perturbation.

TMS was delivered using a single-pulse Magstim 200^2^ stimulator with a double cone coil (Magstim, Whitland, United Kingdom). A skin-tight (swimming) cap was placed on the head of the subject to increase friction between the coil and the scalp. The optimal TMS stimulus site for the right soleus muscle was located on average 1 cm lateral (left) and 1 cm posterior to the cranial apex. Several stimulations were delivered to determine optimal coil placement and it was then marked by a marker pen on the cap. The aMT was defined as the lowest stimulus intensity to elicit clear MEPs in three out of five stimulation from right ankle plantarflexion with 10% IMVC. After the confirmation of aMT, a second swimming cap with a hole in the middle of the vertex (Orca High Visibility Neoprene Swim Cap, Orca, Auckland, New Zealand) was placed over the coil to reduce the gap and relative movement between the coil and head. Then, the custom-made helmet (modified from an ice-hockey helmet; CCM TACK 710 JK-K, CCM Hockey, Montreal, Canada) was attached to the subject’s head with a chin strap. Even though the helmet setup was tight, it was ensured that the helmet was as comfortable as possible with no reported pain caused to the subject. Then subjects moved to the balance system. The TMS cable was placed on a conveyor adjacent to the safety belt conveyor on the roof and connected with the balance platform by a firm handle, which raised the cable above the subject and moved it in the same phase and direction as the balance platform during perturbation (Figure 2). Single-pulse TMS with 110% intensity of aMT was delivered during standing rest and balance perturbation tasks to investigate corticospinal excitability.

For H-reflex measurements, subjects stood relaxed during the electrical stimulation set-up. Electrical stimulation was administrated to the tibial nerve in the popliteal fossa. A cathode (1.5 cm × 1.5 cm) was placed over the tibial nerve, and an anode (5 cm × 8 cm) was placed above the patella. Rectangular stimulation pulse (DS7AH, Digitimer Ltd., Hertfordshire, United Kingdom) with a duration of 0.2 ms was delivered at 10 s intervals. Once the optimal site of stimulation was established, the site was marked by a marker pen, and an electrode (Blue Sensor, Ag/AgCl, 28 mm^2^, Ambu A/S, Ballerup, Denmark) was placed and strapped around the subject’s knee with an elastic band. An increasing intensity interval (1–5 mA) was chosen to measure the H-M recruitment curve with at least 30 data points up to the maximal M-wave. The stimulus intensity was adjusted to 5% (±2%) of the maximum M-wave, which was used during balance perturbation to control H-reflex measurements.

### Dynamic balance perturbations with TMS and H-reflex

Balance perturbation tasks utilized a custom-built dynamic balance device (University of Jyväskylä, Jyväskylä, Finland) modified from Piirainen et al.’s study (2013). The balance perturbation system operated at 0.25 m/s, accelerating at 2.5 m/s^2^ over a 0.3 m displacement. During balance perturbation tasks, 16 balance perturbations were delivered in anterior (plate moved forward) and posterior (plate moved backward) directions in random order with 6–12 s intervals. A fixation point was set on the wall 3 m from the subjects at eye level to stabilize the subjects’ visual attention during measurements.

During balance perturbation tasks, the COP displacement and velocity in anterior and posterior (AP) directions were collected by custom designed balance platform, with one strain gauge sensor in each of the four corners of the force plate (BT4 balance platform; HUR Labs, Tampere, Finland) and saved and analyzed using the Coachtech-feedback system (University of Jyväskylä, Jyväskylä, Finland). COP in anterior-posterior direction was calculated using the formula COPy=((Frr+Frf)×0.26−(Frr+Flr)×0.26)/(Flf+Frf+Frr+Flr) , where lf = left front, rf = right front, rr = right rear, lr = left rear and 0.26 m is sensor distances from middle line.

**FIGURE 3:**
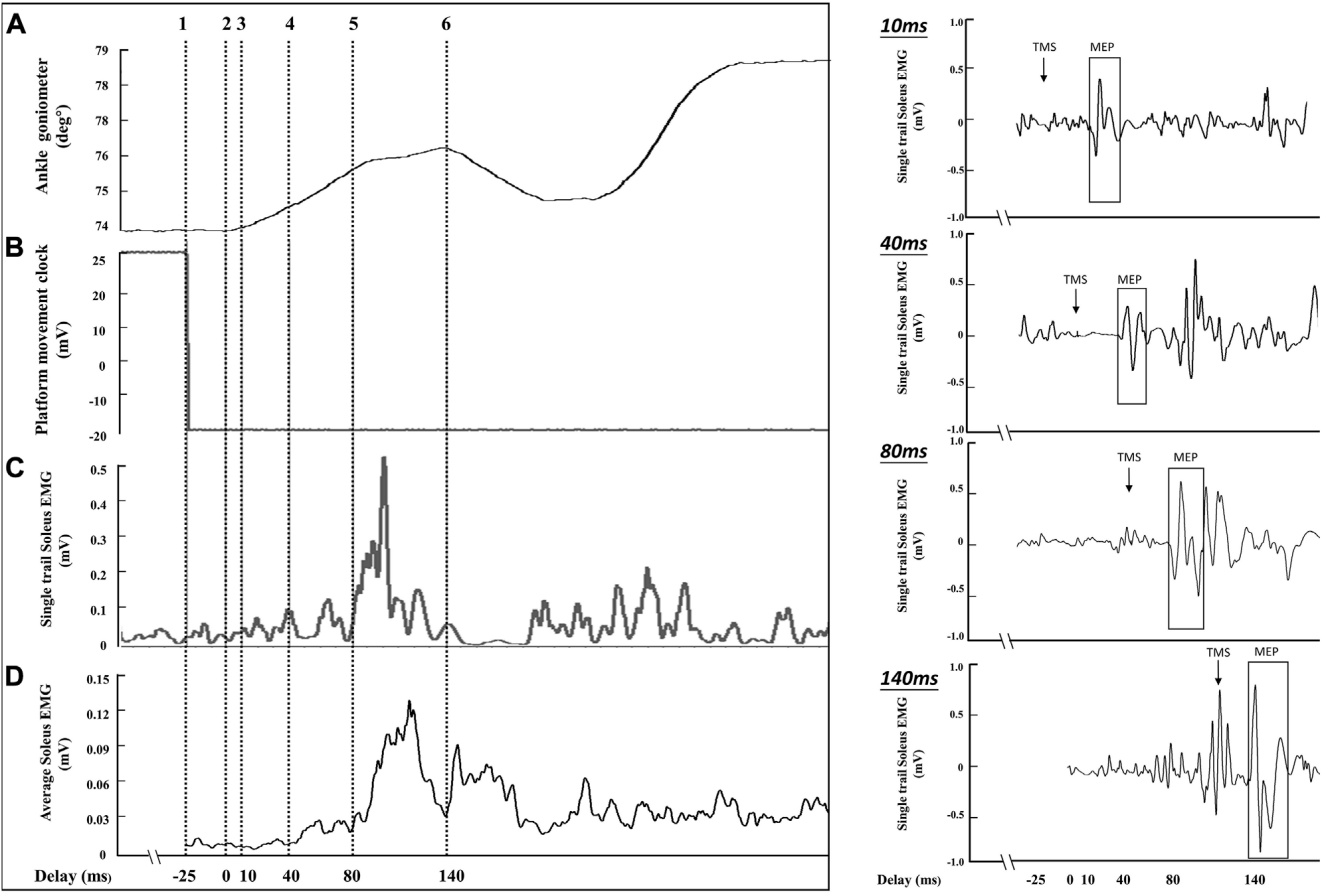
The sampling schematic of one balance perturbation phase. The **(A)** channel shows ankle movement from the ankle goniometer (°), and cursor 2 was determined as the onset of ankle movement after balance perturbation. The **(B)** channel demonstrates platform movement starting from cursor 1. The **(C)** channel shows soleus EMG activities from a single perturbation trial (smoothed with a 2 ms window and rectified). The **(D)** channel shows the average EMG activity curve from 8 posterior perturbation trials (smoothed with a 2 ms window and rectified) to estimate the delay for stimulation because of the EMG variability between perturbation trials. The right part of the figure shows the soleus MEPs from a single trial of perturbation in four delays (10 ms, 40 ms, 80 ms, and 140 ms).

In the pilot study, the time difference between ankle movement identified by the ankle goniometer (Figure 3A: cursor 2) and platform control signal (Figure 3B: cursor 1) was analyzed. A 17 ms–33 ms time difference was observed between ankle movement (cursor 1) and the platform control signal (cursor 2). Therefore, a 25 ms constant delay was defined as the time difference between the platform control signal and the onset of ankle movement. During the balance perturbation task, MEPs and H-reflexes were elicited at four delays after the platform control signal: 35 ms, 65 ms, 105 ms, and 165 ms. Delays of MEPs and H-reflex’s in this study were represented as 10 ms, 40 ms, 80 ms, and 140 ms, using the onset of the ankle movement as the delay timepoint (see Figure 3: cursor 3, 4, 5, and 6). Delays were designed to represent the onset of ankle movement, SLR, LLR, and the voluntary activation phase. Using the same protocol as TMS trials, H-reflex was measured in standing rest and the same four delays during balance perturbation. The maximum compound action potential (M-max) of soleus muscle with was recorded in order to normalize the muscle response values (MEP, H-reflex, and voluntary EMG activity).

In the dynamic balance perturbation tasks with stimulation, 16 perturbations were performed in one set of trials, with 8 anterior and 8 posterior perturbations in random order, which ensured subjects were not able to anticipate the direction of perturbation. Two-min rest periods were given after every perturbation set to minimize possible muscle fatigue (Piirainen et al., 2013). During H-reflex balance perturbation trials, a successful trial was defined as an M-wave response of 5% (±2%) M-max value. The intensity of electrical stimulation was adjusted during perturbation trials to obtain at least five successful trials. If less than five successful backward trials in a normal 16-trial perturbation set were achieved, an extra 8-trial balance perturbation set, four backward and four forward, with random order was performed. For each perturbation task, five successful trials were usually completed within 16–24 perturbations (16-trials + 8-trials), followed by 2 minutes of rest.

### Data and statistical analysis

The COP velocity curve was calculated by differentiating the COP curve by using 20 ms windows. Trials were performed at 6–12 s intervals and triggered when COP was at least 1 s within ±5 mm level from zero level. With this approach, the subject was always standing straight without any anticipation for the upcoming perturbation. Peak COP displacement and the average COP velocity were analyzed in the time window of 1 s before platform movement (Preparation-phase; Pre), during platform movement (Active-phase; Act), and 1 s from the end of platform movement (Recovery-phase; Rec).

EMG activity from balance perturbation was collected from the balance perturbation set without stimulation, which was calculated by the root-mean-square (RMS) with a 20 ms window for SOL, TA, and GM during the perturbation from ankle movement (0 ms) to 160 ms. RMS over a 100 ms window was applied before plate movement. All EMG activity data were normalized by maximum RMS with a 20 ms window during balance perturbation and presented %MaxSOL, %MaxTA, and %MaxGM in the results (Piirainen et al., 2013). Background EMG with stimulation trials was analyzed by RMS with a 30 ms window before stimulation and normalized by Mmax of monopolar (MEPs) and bipolar (H-reflex), respectively.

In standing rest, mean soleus MEPs were determined with peak-to-peak amplitude (in mV) from 10 TMS stimulations. Outliers were identified from the ten trials (±2.5 SD of the mean) and removed before analysis (Avenanti et al., 2006). The average MEP latency and duration were calculated in the standing rest condition and then utilized in the balance perturbation condition. The MEP was defined as starting when EMG was above the mean + 2SD level recorded 100 ms before the TMS trigger and ending when below the mean - 2SD level (Hirano et al., 2016). However, this was only used in the standing condition since it was difficult to use these criteria during the perturbation due to increase in EMG. Thus, the MEP amplitude was obtained by calculating the peak-to-peak amplitude within the MEP onset and offset latencies calculated in the standing condition. Selecting MEP amplitudes from 7 - 8 trials when the platform moved backward and averaged after excluding outliers (±2.5 standard deviation of the mean). All MEPs were normalized by the peak-to-peak value of maximum M-wave and presented as %M-max in the results. H-reflex was determined with peak-to-peak amplitude and averaged from all successful trials (within 3%–7% M-max) in standing rest and balance perturbation tasks. H-reflex was normalized by the peak-to-peak amplitude of the maximum M-wave and presented as % M-max in the results.

Statistical analyses were conducted using IBM SPSS 20.0 (SPSS, Chicago, United States). Result visualizations were performed using Prism (V9, GraphPad Software, San Diego, California United States). All variables of MEPs and RMS of EMG activity were processed by log transformation prior to statistical analyses following Nielsen’s suggestion (Nielsen, 1996) since the original data was not normally distributed, which resulted in data being normally distributed as assessed by Shapiro-Wilk’s W tests. Between-session differences for IMVC, TMS intensity of aMT, maximum COP displacement and average COP velocity were examined by paired t-test.

To assess modulation in corticospinal excitability during balance perturbations, MEPs, H-reflex, EMG activity without stimulation, and background EMG before stimulation data were assessed by two-way (2 × 4) repeated-measures ANOVA with the factors SESSION (S1 and S2) and DELAY (10 ms, 40 ms, 80 ms, and 140 ms). When a significant F-value was observed, Mauchly’s test was used to evaluate sphericity, and where the assumption was valid F-values were reported with sphericity-assumed degrees of freedom and df error [i.e., F _(sphericity assumed df, df error)_]. Effect sizes for the ANOVA main effects are reported as partial eta squared (η_p_^2^), where 0.02, 0.13, and 0.26 are considered small, medium, and large, respectively. If significance for DELAY was revealed, Bonferroni post-hoc analysis was used for pairwise comparisons between levels (0 ms, 40 ms, 80 ms, and 140 ms). The significance level was set at *p* < 0.05 and all results were displayed as Mean ± SD.

For the research question of test-retest reliability, a paired t-test was used to test the reliability of log transformed MEP and H-reflex amplitudes between sessions at each delay separately. Test-retest reliability and inter-individual variability of MEPs and H-reflex amplitude between sessions were assessed *via* intraclass correlation coefficients (ICC) using a two-way mixed effects model with an absolute agreement using the average value from multiple trials. Standard error of measurement (SEM) was estimated as root mean square error ( $\sqrt{MSE}$ ) from a one-way ANOVA, which avoids errors associated with ICC calculation. The minimal detectable change (MDC) was calculated as $SEM\times 1.96\times \sqrt{2}$ (Weir, 2005). According to the ICC method guideline (Koo and Li, 2016), ICC was calculated between single stimulation trials, and trial-to-trial coefficient of variance (CV) with homoscedasticity of MEPs and H-reflex amplitudes to determine whether eight MEP/H-reflexes were adequate for calculating the average value. Reliability based on ICCs and 95% CIs were categorized as poor (ICC <0.5), moderate (0.5 < ICC <0.75), good (0.75 < ICC <0.9), or excellent (ICC >0.9). Bland-Altman plots of MEPs in all conditions were investigated to visualize the agreement between two sessions (Bland and Altman, 1995).

## Results

### Motion capture results from pre-study

**FIGURE 4:**
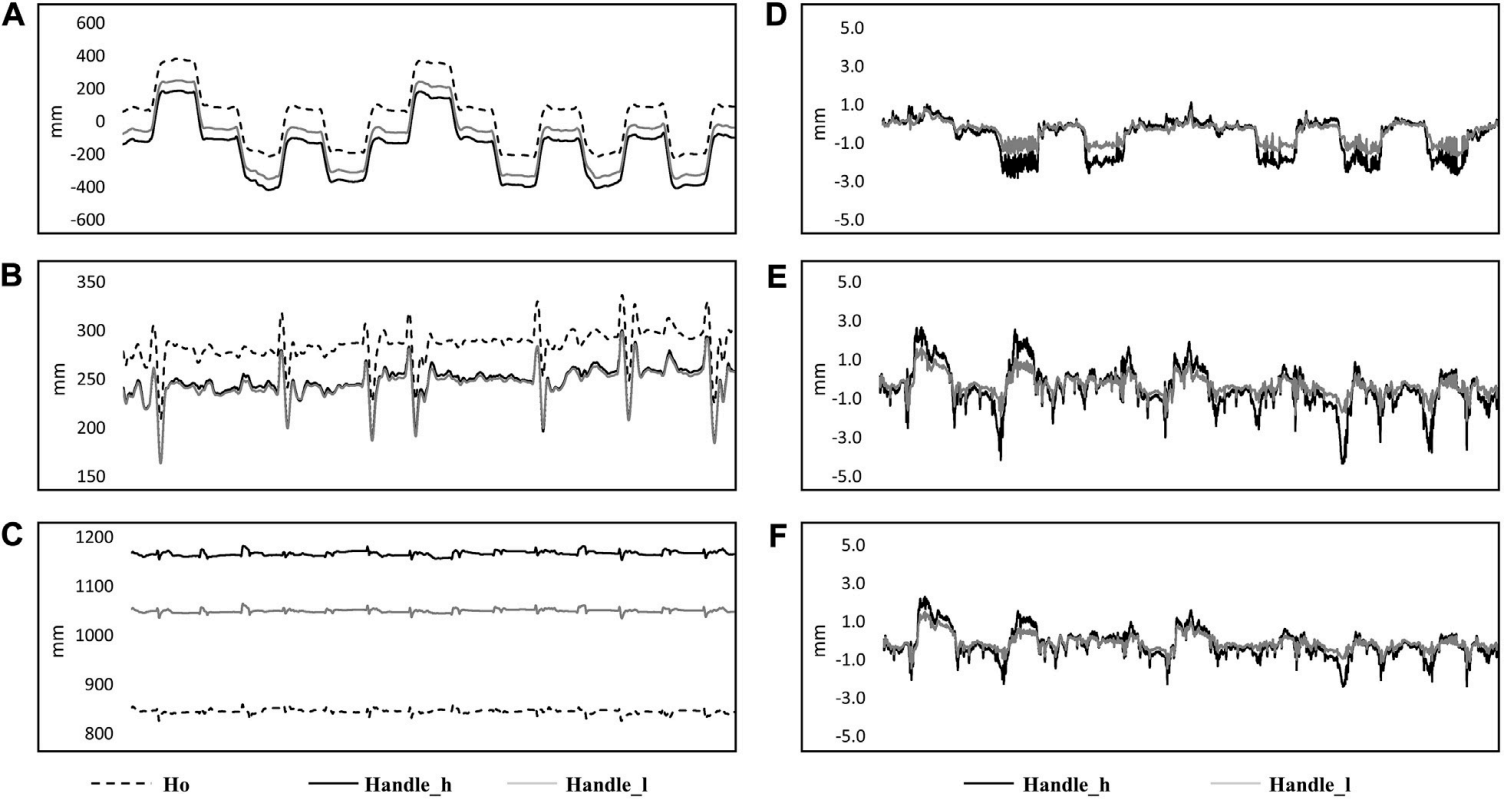
Kinematic data from a single subject in 14 perturbations are shown in the figure. Original data in the ground coordinate system are shown on the left side [**(A)** x-axis; **(B)** y-axis; **(C)** z-axis] with markers on the subject’s head (Ho: dashed line), higher position of the handle (Handle_h: black solid line) and lower position (Handle_l: gray solid line). Coordinate transformed data from markers on the higher position (Handle_l: black solid line) and lower position of the handle (Handle_h: grey solid line) are shown on the right side, which represented the movement of the markers related to the coordinate system of the head [**(D)**: x-axis; **(E)** y-axis; **(F)** z-axis].

Markers of the TMS handle displacement before (A: *x*-axis, B: *y*-axis, and C: *z*-axis) and after transformation (D: *x*-axis, E: *y*-axis, and F: *z*-axis) are shown in Figure 4. The maximum offset of the marker on the higher position of the TMS handle demonstrated 7 ± 2 mm in the *x*-axis, 8 ± 2 mm in the *y*-axis, and 5 ± 1 mm in the *z*-axis. The offset of the marker on the lower position of the TMS handle was 5 ± 1 mm in the *x*-axis, 5 ± 2 mm in the *y*-axis, and 4 ± 1 mm in the *z*-axis.

### Between-session test-retest reliability

The peak-to-peak amplitude of MEPs and H-reflexes varied from 0.87 ± 0.61 to 2.51 ± 1.47 mV and from 1.54 ± 0.64 to 3.20 ± 1.68 mV, respectively. In addition, MEPs were visible in 100% of the trials. By paired-t test, MEPs demonstrate lower amplitude in rest standing [t_(13)_ = 2.217, *p* = 0.045, η^2^ = 0.592] and 10 ms delay (t_(13)_ = 2.211, *p* = 0.046, η^2^ = 0.591) in the perturbation task of S2. No significant difference was demonstrated for MEP amplitude in other delays of the perturbation tasks [40 ms: t_(13)_ = 1.455, *p* = 0.169, η^2^ = 0.389; 80 ms: t_(13)_ = 0.561, *p* = 0.585, η^2^ = 0.150; 140 ms: t_(13)_ = 0.946, *p* = 0.361, η^2^ = 0.253]. The H-reflex increased in S2 compared to S1 at 10 ms delay [t_(12)_ = −2.460, *p* = 0.03, η^2^ = −0.682], but there were no differences in the other conditions [standing rest: t_(12)_ = −0.720, *p* = 0.486, η^2^ = −0.200; 40 ms: t_(12)_ = −0.765, *p* = 0.459, η^2^ = −0.212; 80 ms: t_(12)_ = −0.973, *p* = 0.350, η^2^ = −0.270; 140 ms: t_(12)_ = −1.303, *p* = 0.217, η^2^ = −0.362].

**FIGURE 5:**
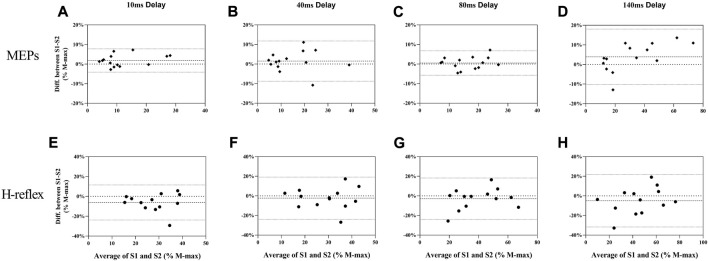
Bland-Altman plot for MEPs **(A**–**D)** and H-reflex **(E**–**H)** responses in balance perturbation between S1 and S2. Each panel (10 ms, 40 ms, 80 ms, and 140 ms) shows the difference as a function of the average of the two testing sessions with dashed lines indicating the mean bias and 95% confidence intervals indicated by dot lines.

MEPs during standing rest demonstrated excellent test-retest reliability between sessions (ICC = 0.932; Table 1) when considering the 95% CIs. During balance perturbation tasks, MEPs also showed excellent reliability (ICC = 0.928–0.947; Table 1). From the Bland-Altman plot, the mean bias for MEPs at 10 ms delay (Figure 5A, mean bias = 1.85%, 95%CI [−4.09%, 7.80%]) and 40 ms delay (Figure 5B, mean bias = 1.50%, 95%CI [−8.78%, 11.79%]) were similar. MEPs at 80 ms delay showed the lowest bias (Figure 5C, mean bias = 0.50%, 95%CI [−5.76%, 6.77%]), while MEPs of 140 ms delay demonstrated the highest bias and widest limits of agreement (Figure 5D, mean bias = 3.91%, 95%CI [−10.23%, 18.04%]).

**TABLE 1:** Between-session test-retest reliability of MEPs (log-transformed data) and H-reflex (original data) with ICC and 95% confidence intervals. In H-reflex, SEM/MDC is expressed in decimal form, which is the same as the original H-reflex data.

	MEPs			H-reflex		
	ICC [95%CI]	SEM	MDC	ICC [95%CI]	SEM(%Mmax)	MDC (%Mmax)
Standing rest	0.932 [0.789, 0.978]	0.232	0.644	0.475 [−0.771, 0.841]	0.071	0.169
10 ms delay	0.935 [0.811, 0.979]	0.210	0.581	0.626 [−0.079, 0.881]	0.158	0.378
40 ms delay	0.928 [0.797, 0.977]	0.152	0.420	0.720 [0.086, 0.914]	0.063	0.151
80 ms delay	0.943 [0.777, 0.982]	0.032	0.088	0.887 [0.644, 0.965]	0.071	0.169
140 ms delay	0.947 [0.835, 0.983]	0.084	0.232	0.865 [0.577, 0.958]	0.126	0.302

During standing rest, H-reflex demonstrated poor test-retest reliability (ICC = 0.475; Table 1). During balance perturbation tasks, H-reflex showed moderate-to-good reliability (ICC = 0.626–0.887). At 10 ms delay, ICC demonstrated a wider 95% CI [−0.079, 0.881] compared to the other delays. Meanwhile, H-reflex showed highest bias at the 10 ms delay (Figure 5E, mean bias = −6.149%, 95%CI [−23.81%, 11.51%]). Similar limits of agreement for H-reflex were observed at 40 ms (Figure 5F, mean bias = −2.328%, 95%CI [−23.82%, 19.17%]) and 80 ms delays (Figure 5G, mean bias = −2.909%, 95%CI [−24.02%, 18.21%]). However, H-reflex demonstrated its widest limits of agreement at the 140 ms delay (Figure 5H, mean bias = −4.935, 95%CI [−31.68%, 21.81%]).

### Within-session test-retest reliability

In S1, ICC of MEPs showed excellent reliability and narrow 95% CI in standing rest and balance perturbations (ICC = 0.927–0.983). In S2, ICC demonstrated good to excellent reliability of all MEPs (ICC = 0.854–0.976). Within-session CV% of MEPs ranged from 20.1% to 41.6% in both sessions and showed homoscedasticity when tested by Levene’s statistics (Table 2).

**TABLE 2:** Within-session test-retest reliability (between stimulation trials) of MEPs for S1 and S2 are shown in the table with ICC and 95% confidence interval. CV% was shown as mean ± sd.

	S1		S2	
	ICC [95%CI]	CV%	ICC [95%CI]	CV%
Standing rest	0.953 [0.906, 0.982]	40.8 ± 14.5	0.934 [0.868, 0.975]	39.4 ± 11.7
10 ms delay	0.927 [0.932, 0.993]	39.1 ± 25.8	0.915 [0.924, 0.987]	41.6 ± 17.5
40 ms delay	0.964 [0.925, 0.987]	39.0 ± 23.1	0.960 [0.913, 0.986]	38.5 ± 20.7
80 ms delay	0.957 [0.909, 0.985]	33.4 ± 17.8	0.854 [0.694, 0.948]	35.0 ± 14.5
140 ms delay	0.983 [0.964, 0.994]	20.1 ± 8.0	0.976 [0.950, 0.991]	22.7 ± 10.3

Within-session reliability of H-reflex responses showed to be good to excellent in both sessions (ICC = 0.874–0.994), and narrow 95% CI. Within-session CV% of H-reflex was 16.9–33.1% in both sessions and Levene’s test indicated homoscedasticity (Table 3).

**TABLE 3:** Within-session test-retest reliability (between stimulation trials) of H-reflex for S1 and S2 are shown in the table with ICC and 95% confidence interval. CV% was shown as mean ± sd.

	S1		S2	
	ICC [95%CI]	CV%	ICC [95%CI]	CV%
Standing rest	0.985 [0.968, 0.995]	21.5 ± 9.8	0.986 [0.971, 0.995]	18.6 ± 5.9
10 ms delay	0.945 [0.848, 0.989]	27.9 ± 11.8	0.956 [0.725, 1.000]	22.3 ± 8.0
40 ms delay	0.945 [0.837, 0.991]	33.1 ± 12.7	0.994 [0.963, 1.000]	22.1 ± 9.8
80 ms delay	0.979 [0.905, 0.999]	19.1 ± 11.7	0.874 [0.351, 0.997]	16.9 ± 8.6
140 ms delay	0.974 [0.881, 0.999]	24.0 ± 16.7	0.965 [0.843, 0.999]	17.8 ± 8.3

### COP in balance perturbation

**FIGURE 6:**
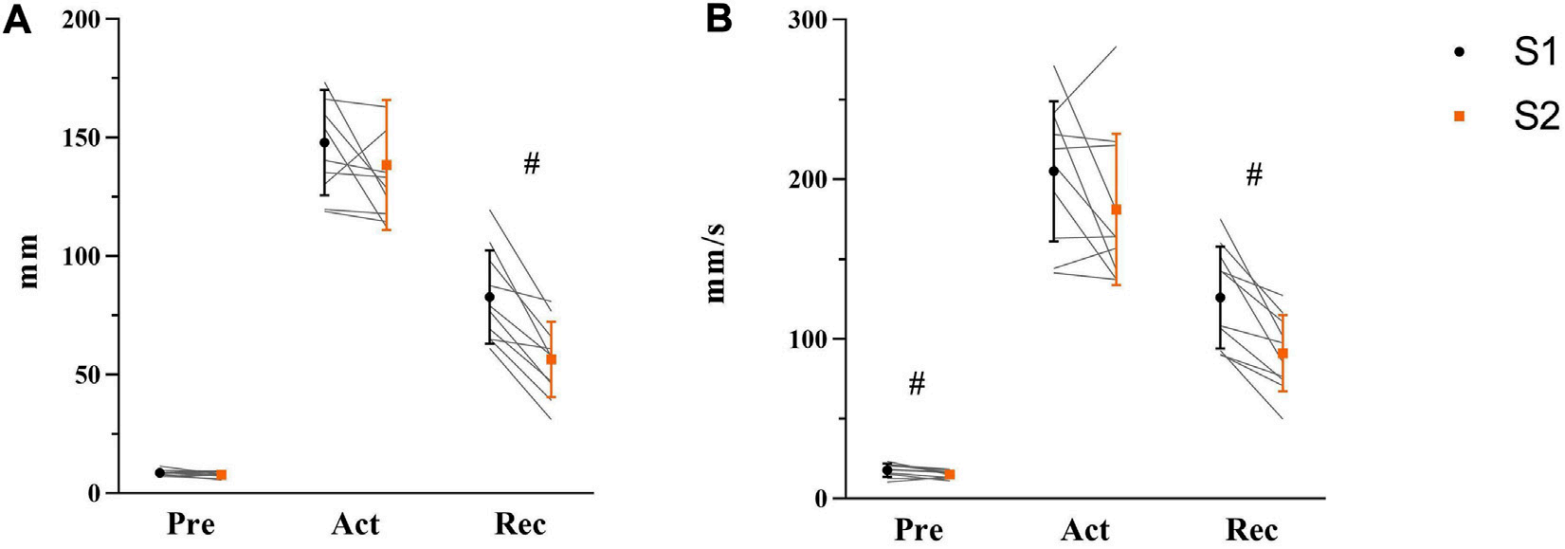
Maximum COP displacement **(A)** and average COP velocity **(B)** in Pre-, Act-, and Rec-phases were shown respectively with mean, standard deviation, and individual data (light gray line) in the figure (S1: black symbol; S2: orange symbol, N = 10). Significant differences were marked by ‘#’ between sessions (*p* < 0.05).

COP displacement and velocity of Pre-, Act-, and Rec-phases were analyzed to explore the balance performance in AP direction before, during, and after of balance platform moving, respectively (see Figure 6). Before perturbation (Pre), paired t-test results indicated no change in maximum COP displacement from S1 to S2 [t_(9)_ = 1.665, *p* = 0.132, η^2^ = 0.235]. However, velocity was lower in S2 (15 ± 2 mm/s) than S1 (18 ± 4 mm/s) [t_(9)_ = 2.817, *p* = 0.020, η^2^ = 0.469]. During perturbation (Act), there was no difference shown either in COP displacement [t_(9)_ = 1.247, *p* = 0.244, η^2^ = 0.147] or velocity (t_(9)_ = 1.650, *p* = 0.133, η^2^ = 0.232). After perturbation (Rec), significant differences between S1 and S2 were demonstrated from both COP displacement [S1: 83 ± 20 mm; S2: 56 ± 16 mm, t_(9)_ = 5.962, *p* < 0.001, η^2^ = 0.798] and velocity [S1: 126 ± 32 mm/s; S2: 91 ± 24 mm/s, t_(9)_ = 5.043, *p* = 0.001, η^2^ = 0.739].

### EMG activity during balance perturbation

**FIGURE 7:**
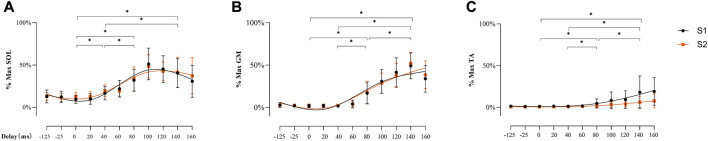
EMG activity of SOL **(A)**, GM **(B)**, and TA **(C)** during balance perturbation respectively Significant differences between delays were marked by ‘*’ (*p* < 0.05). EMG activity was demonstrated with mean and standard deviation. The line was processed by smoothing the spline of data knots for S1 (black) and S2 (orange).

There was no main effect of soleus muscle EMG activity for SESSION [F _(1, 26)_ = 0.128, *p* = 0.723, η_p_^2^ = 0.005], but a significant main effect was demonstrated for DELAY [Figures 7A,F_(3.326, 60.485)_ = 65.839, *p* < 0.001, η_p_^2^ = 0.718]. At the delays studied, post-hoc analyses showed lower EMG activity at 10 ms delay than 40 ms, 80 ms, and 140 ms (all *p* < 0.001). At 40 ms delay, EMG activity was lower than 80 ms and 140 ms respectively (both *p* < 0.001), but there was no difference between 80 ms and 140 ms (*p* = 0.706).

Similarly, there was no main effect observed in gastrocnemius medial muscle EMG activity for SESSION [F _(1, 26)_ = 1.513, *p* = 0.230, η_p_^2^ = 0.055], but a significant main effect was observed for DELAY [Figures 7B,F_(2.077, 54.009)_ = 219.095, *p* < 0.001, η_p_^2^ = 0.894]. Specifically, post-hoc analysis showed lower EMG activity at 10 ms delay than 80 ms, and 140 ms (both *p* < 0.001), and EMG activity at 40 ms delay was lower compare with 80 ms and 140 ms (both *p* < 0.001). Significantly lower EMG activity was also observed at 80 ms than 140 ms (*p* < 0.001).

The EMG activity of tibialis anterior muscle demonstrated no main effect for SESSION (F _(1, 26)_ = 3.488, *p* = 0.073, η_p_^2^ = 0.118), but significant main effect for DELAY [Figures 7C,F_(2.194, 57.045)_ = 122.897, *p* < 0.001, η_p_^2^ = 0.825]. Specifically, post-hoc analyses showed lower EMG activity at 10 ms delay than 80 ms, and 140 ms (both *p* < 0.001). EMG activity at 40 ms was lower compared with 80 ms and 140 ms (both *p* < 0.001), and significantly lower EMG activity was observed at 80 ms than 140 ms (*p* < 0.001).

The background EMG before TMS did not differ between sessions [F _(1, 26)_ = 0.317, *p* = 0.578, η_p_^2^ = 0.12], but significant increases were observed between 140 ms delay (1.72%) with other delays (10 ms: 0.53%, *p* < 0.001; 40 ms: 0.50%, *p* < 0.001; 80 ms: 0.58%, *p* < 0.001). A significant difference was also found between 40 ms and 80 ms delays (*p* = 0.014). Background EMG before electrical stimulation has shown similar results. No difference between sessions (F _(1, 24)_ = 0.383, *p* = 0.542, η_p_^2^ = 0.016). Compared to other delays (10 ms: 0.44%, *p* < 0.0001, 40 ms: 0.48%, *p* < 0.0001, 80 ms: 0.46%, *p* < 0.0001), background EMG at 140 ms delay was significantly higher (1.02%).

### Corticospinal excitability during balance perturbation

There was no difference observed in IMVC (S1: 1814.6 ± 499 Nm, S2: 1871.9 ± 522 Nm, *p* = 0.894) or TMS intensity of aMT (S1: 35% ± 4%, S2: 35% ± 4%, *p* = 0.769) between sessions.

**FIGURE 8:**
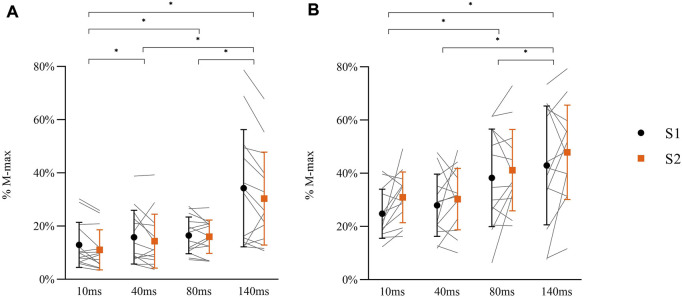
Peak-to-peak amplitude of MEPs (**A**: N = 14) and H-reflex responses (**B**: N = 13) in balance perturbation with delays (10 ms, 40 ms, 80 ms, 140 ms) from two sessions (S1: black symbol, S2: orange symbol). Data shown are mean, standard deviation, and individual data (light gray line). The significant differences are shown between delays by ‘*’ (*p* < 0.05), but there were no between-session differences in MEPs or H-reflex.

A significant main effect for DELAY in MEPs during balance perturbation was observed [Figures 8A,F_(3, 78)_ = 56.764, *p* < 0.001, η_p_^2^ = 0.686], while no changes were shown between sessions [F _(1, 26)_ = 0.033, *p* = 0.858, η_p_^2^ = 0.001]. Post-hoc analyses demonstrated significant lower MEPs at 10 ms compared with other delays (40 ms: *p* = 0.009; 80 ms: *p* = 0.001; 140 ms: *p* < 0.001). MEPs at 140 ms delay were higher than 40 ms and 80 ms delays (both *p* < 0.001), but no differences were observed between 40 ms delay and 80 ms delay (*p* = 0.249) (Figure 8A).

A main effect for DELAY was observed in H-reflex [Figures 8B,F_(1.594, 38.249)_ = 19.366, *p* < 0.001, η_p_^2^ = 0.447], while no differences between sessions were observed [F _(1, 24)_ = 0.692, *p* = 0.414, η_p_^2^ = 0.028]. Post-hoc analyses demonstrated that H-reflex at 10 ms was lower than 80 ms (*p* = 0.001) and 140 ms (*p* < 0.001) delays. Lower H-reflex was also shown at 40 ms delay compared to 80 ms and 140 ms (80 ms: *p* = 0.001, 140 ms: *p* = 0.001). There was no difference between 80 ms and 140 ms delay (*p* = 0.172) (Figure 8B).

## Discussion

In the present study, we investigated the reliability of corticospinal (MEPs) and spinal excitability (H-reflex) during balance perturbation, using variances estimated from a two-session test-retest paradigm. At the beginning of the balance perturbation phase (10 ms delay), MEPs and H-reflexes demonstrated a significant difference between sessions assessed by paired t-test. ICC demonstrated good-to-excellent test-retest reliability in the TMS measurements, which was generally better than that of the H-reflex measurements. Within each session, both measurements showed excellent reliability, although variability was also shown between trials. No differences between the sessions were observed in MEPs, H-reflex responses, COP displacement, or COP velocity during balance perturbations indicating good reliability of the test methods. Neither EMG activity without stimulation nor background activity before stimulation demonstrated changes between sessions, indicating constant muscle activity between measurement sessions.

### Test-retest reliability of the experiment method

In the pre-study, the coil and its handle were considered as a rigid body, which rotated around the head as the center. Two markers on the upper and lower part of the handle were used to estimate the movement of the coil. Because the coil was below the lower marker, the movement of the coil could be considered to be less than the markers on the handle, which was less than 5 mm in the *x-, y-,* and *z-*axis. From the study of TMS coil location accuracy with a function-guided navigation system, 2 mm–5 mm distance around the initially defined hotspot resulted in good accuracy of MEPs, and changes in coil location within 5 mm distance had no significant effect on MEP amplitude (De Goede et al., 2018). This supports our assertion that the stability of the TMS coil during balance perturbation trials provided accurate MEP values in the present study.

Paired t-tests were used to test any systematic differences in MEPs and H-reflexes between sessions in this study. According to paired t-test results, 10 ms delay of S2 resulted in higher H-reflex amplitude (S1: 24.8% Mmax; S2: 31.0% Mmax) with lower MEP (S1: 13.0% Mmax; S2: 11.1% Mmax). The observed MEP or H-reflex amplitude changes were lower than the between-session MDC, which indicates that the between session differences may result from the variability of MEPs/H-reflex or noise in the measurements. Therefore, data should be interpreted carefully because systemic error may occur in some conditions. It would be recommended that at least two familiarizing perturbation sets should be performed before the first measurement session to reduce possible learning effects.

TMS measurement demonstrated strong test-retest reliability, both between- and within-session during standing rest and balance perturbation tasks (ICC >0.80). The highest test-retest reliability and lowest between-trial variability were observed at 140 ms delay, which is defined as a voluntary activation phase in the present study. It indicates that MEPs are more reliable while the contribution of voluntary activation of the muscles is increasing compared with the low voluntary muscle activity at the early response phases after balance perturbation or muscles at rest. This finding is supported by Tallent et al.’s (2012) study, in which they showed higher reliability of MEPs in active muscle than resting muscle. In Darling et al.’s (2006) study, less variance was also observed with more muscle activation. Sensory inputs (vestibular, vision, proprioception) may influence the excitability of motor units in the corticospinal pathway more at standing rest and early phases after perturbation and, therefore, the variability of MEPs increases (Darling et al., 2006). Another reason, such as intersession intervals (>72 h), would reduce the TMS measurement reliability (Luc et al., 2014; Cavaleri et al., 2017). There may be a reason for the good between-session (<53 h) MEPs reliability in this study. Examine the mean MEP value from several individual trials because of typical between-trial variability, which was also shown in this study (within-session CV% = 16.9%–46.1%). Although Goldsworthy et al. (2016) suggested that 20–30 trials may be optimal for estimating MEPs in the first dorsal interosseous by TMS, other TMS studies have also shown good reliability with fewer stimulation trials (Van Hedel et al., 2007; Bastani and Jaberzadeh, 2012), which indicates that the reliability of MEPs fluctuates in different experimental protocols and it might be muscle specific (Cavaleri et al., 2017). MEPs in lower limb muscles, on the other hand, appear to be more reliable than those in upper limb muscles. For example, eight to ten trials of MEPs showed excellent reliability (ICC >0.81) in the tibias anterior muscle of stroke patients (Beaulieu et al., 2017). In addition, Lewis et al.‘s (2014) demonstrated good reliability (ICC >0.80) in soleus muscle in healthy subjects by averaging only six MEPs. According to Cavaleri et al.’s (2017) study, a mean value of ten trials is required to produce consistent condensed reliability, and five trials are the lowest number to achieve excellent within-session reliability. In the present study, MEPs of a single subject at every delay were analyzed from 8 backward balance perturbation trials and the average value was calculated (7 – 8 trials) after removing outliers. To the best of our knowledge, there is only one previous TMS study that has used this method (Hosel and Tremblay, 2021). Since ICC of MEPs demonstrated good-to-excellent within-session (between trials) reliability, calculating average MEP amplitude from 8 TMS stimulation trials and removing outlier MEPs beyond 2.5 SD (maximum one outlier in the present results) could be considered as sufficient in reducing MEP between-trial variability and producing a reliable TMS procedure in corresponding balance perturbation tasks.

H-reflex demonstrated better test-retest reliability in balance perturbation task than at standing rest; ICC, SEM, and MDC, and within-session reliability were extremely robust. Similar results that revealed high stability between stimulation trials but lower reliability between the sessions were found in a previous study (Handcock et al., 2001). The possible reasons include more irregular body sway or various lack of attention issues during standing rest compared with more regular body movements and better focus during balance perturbation. Compared with the standing position, previous studies with subjects who were in supine or prone position revealed high reliability for the soleus H-reflex (Hopkins et al., 2000; Palmieri et al., 2002), which indicated that the H-reflex reliability may relate to the body position used in the protocol. Better reliability was shown at 80 ms (ICC = 0.89) and 140 ms (ICC = 0.87) delays than at 10 ms (ICC = 0.63) and 40 ms (ICC = 0.72) delays, even though the reliability in the latter two conditions are still acceptable (Portney and Watkins, 2009). The within-session reliability was generally better than between sessions in the present study. This observation implies that five successful stimulations, i.e., at the range of 3%–7% M-max, is sufficient to be utilized for H-reflex measurements in balance perturbation tasks. The average of a larger number of trials (8–10) may provide greater reliability between the sessions. However, it should be noted that in this kind of protocol, the number of perturbation trials will increase with increasing stimulation responses, which might increase the risk of fatigue. It is not surprising that high reliability of H-reflex in soleus muscle was shown between stimulation trials, since previous studies from different body positions have also reported similar high reliability values, and, thus suggested that 4 to 5 stimulations are needed to obtain reliable results (Hopkins et al., 2000; Al Amer et al., 2020). Although the stimulation intensity and body position were different in this study, the present study adds important information about reliability of using the H-reflex method in dynamic balance perturbation tasks.

### Corticospinal modulation in balance perturbation

During balance perturbation tasks, COP is an important parameter to evaluate balance performance (Zemková, 2011). In a previous study of balance ability between young and older adults, the older subjects showed larger peak COP displacement which implied poor balance control ability during perturbation (Piirainen et al., 2013). In the present study, we were more interested in the AP direction of the body sway, thus the COP displacement and velocity were analyzed only to backward movement of the platform. COP displacement and velocity did not differ significantly between the two measurement sessions in terms of Act-phase, indicating high reliability of COP during the active balance perturbation phase between sessions. However, the velocity of COP during Pre- and Rec-phases, as well as the maximum COP displacement during Rec-phase was considerably reduced in S2. The results suggest that there was less body sway before perturbation began and after perturbation ended in S2, which may indicate effects of learning. Nevertheless, these changes were not observed during the Act-phase when stimulations were delivered.

As we already know, a rapid ankle joint perturbation (dorsiflexion) can lead to a relatively stereotypical pattern response around 40 ms in the soleus muscle, which is addressed as the ‘SLR’. When H-reflex was produced at this time, it showed facilitation in a previous drop jump study, which was explained by enhanced Ia-afferent transmission (Taube et al., 2008). However, H-reflex responses in the present results did not show any difference between the 10 ms delay and 40 ms delay, which was similar to the case of Piirainen et al.’s study (Piirainen et al., 2013). It may relate to the different ankle movement patterns between balance perturbation (translation) and drop jump (rotation). As demonstrated by Wälchli et al.’s study, it involved higher speed perturbations (0.74 m/s), in which SLR decreased while the LLR increased, inferring a top-down control from supraspinal sources (Wälchli et al., 2017). MEP amplitudes were slightly enhanced at 40 ms with EMG activity of the soleus muscle increasing from the onset of ankle movement (no stimulation trials) but background EMG before stimulation did not change. Meanwhile, gastrocnemius and tibialis anterior muscles have not been active implying no co-contraction of the agonist-antagonist muscle groups at this delay (see Figure 7). As a result of the present findings, it seems that cortical control contributes to the initial phase following perturbation. However, there is no literature demonstrated that the transcortical loop triggers the early phase after perturbation. Therefore, the enhanced MEP may relate to extra caution during perturbation tasks.

H-reflexes were found to be enhanced from SLR to LLR during balance perturbation in Taube et al.’s previous study (Taube et al., 2006), suggesting that the LLR is part of the transcortical loop in the soleus muscle. The present results did not show a significant difference between 40 ms (SLR) to 80 ms (LLR) delay but between 10 ms and 80 ms/140 ms delay, which is not entirely consistent, but not in conflict with Taube’s study, since both studies indicate an increase in H-reflex during balance perturbation at the later phase. It is also important to consider the random direction of perturbation in this study, as well as different speeds and displacements of the balance platform movement. Therefore, direct comparison is not possible. There was a significant increase in background EMG levels (before stimulation) at 140 ms delay. Increased voluntary muscle contractions result in increased MEP and H-reflex values (Škarabot et al., 2019), which may explain the increased MEPs and H-reflexes in the muscle voluntary contraction phase during perturbation tasks.

### Limitations

Some study limitations should be considered when interpreting the current findings. We did not use a neuronavigation TMS system in the present study. However, it is very difficult to utilize such a system in the dynamic task and with the helmet used in the current experiment. The helmet and the coil conveyor made it possible to stabilize the coil during the experiment and eliminate the tension of the cable during perturbations. The pre-study on the stability of the helmet system only included two subjects, which did not provide any statistical results. On the other hand, both subjects showed relatively small movement of the coil, which is in line with the literature (De Goede et al., 2018). The small sample size in this study is another limitation and since MEPs (pseudo-monopolar) and background EMG (bipolar) used different arrangement normalizing MEPs by background EMG is complicated. Therefore, we were only able to discuss the corticospinal modulation and changes in background EMG separately.

## Conclusion

In conclusion, this study investigated the reliability of TMS and H-reflex measurements during different phases of a sliding-platform balance perturbation task. The TMS coil stability was verified in the pre-study experiment with kinematic data. Both MEPs and H-reflex demonstrated acceptable reliability between two measurement sessions based on ICCs and have good-to-excellent test-retest reliability between stimulation trials. However, careful placement/stability of the coil and control of the M-wave during dynamic balance perturbation trials must be ensured to obtain such reliable data. MEPs increased in the early phase (SLR) implying that the corticospinal loop may play a role in overcoming balance perturbation at an earlier delay than previously thought.

## Data Availability

The raw data supporting the conclusions of this article will be made available by the authors, without undue reservation.
